# Supplementary material for: High-dose naloxone: Effects by late administration on pain and hyperalgesia following a human heat injury model. A randomized, double-blind, placebo-controlled, crossover trial with an enriched enrollment design
Source: PLoS One. 2020 Nov 12;15(11):e0242169. doi: 10.1371/journal.pone.0242169 (PMC7660513; doi:10.1371/journal.pone.0242169)
Supplement: S1 Protocol — (PDF) [file pone.0242169.s003.pdf]

PROTOCOL

Effect of High-dose Target-controlled Naloxone Infusion on Pain and Hyperalgesia in Patients following Recovery from Impacted Mandibular Third Molar Extraction.

A Randomized, Placebo-controlled, Double-blind Crossover Study

This protocol is based on two previous high-dose naloxone studies, one completed and one ongoing, approved of the Regional Committee of Research Ethics of the Capital Region (H-2-2012-174; H-15002712), the Danish Health and Medicines Agency (2012-005663-17; 2015-000793-36) and the Danish Data Protection Agency (2013-41-1475; RH-2014-30-1150, I-Suite nr.: 02815).

CONTENT:

TITLE PAGE 1
CONTENT 2
NAME AND CONTACT INFORMATION OF THE INVESTIGATORS 4
SPONSOR'S AND PRINCIPAL INVESTIGATOR'S SIGNATURES 5
1. INTRODUCTION AND RATIONALE OF THE STUDY 6
1.1 Background
1.2 Reference list
2. AIM 9
3. OUTCOMES 9
3.1 Primary Outcomes
3.1.1 Mandibular third molar extraction study
3.1.2 Burn injury study
3.2 Secondary Outcomes
3.2.1 Mandibular third molar extraction (TME) study
3.2.2 Burn injury (BI) study
4. ETHICAL CONSIDERATIONS 10
4.1 General ethical considerations
4.2 Risks and inconveniences for the study subjects
4.2.1 Study drug (all participants)
4.2.1.1 Acute Effects
4.2.1.2 Protracted Effects
4.2.1.3 Conclusion
4.2.2 Burn injury (BI-participants)
4.2.2.1 Adverse effects
4.2.2.2 Conclusion
4.3 Informed Consent
4.3.1 Impacted mandibular third molar extraction study (TME-participants)
4.3.2 Burn injury study (BI-participants)
4.4 Data Registration and Protection
5. TIMELINE AND LOCATION OF THE STUDY 13
5.1 Estimated time plan (all participants)
5.2 Duration of examination days
5.2.1 TME-participants
5.2.2 BI-participants
5.3 Location (all participants)
6. STUDY DESIGN 13
6.1 TME-participants
6.2 BI-participants
7. SELECTION CRITERIA 13
7.1 Inclusion Criteria
7.1.1 TME-participants
7.1.2 BI-participants
7.2 Exclusion Criteria
7.2.1 TME-participants
7.2.2 BI-participants
8. PRACTICAL CONSIDERATIONS 16
8.1 Economical issues
8.2 Participant inclusion and withdrawal from the study
8.3 Reasons to withdraw a participant from the study
8.4 Procedures for participants withdrawing from the study
9. METHODS 16
9.1 Study algorithms
9.1.1 TME-study
9.1.1.1 Day 1/Day 2
9.1.2 BI-study
9.1.2.1 Screening day: Day 0
9.1.2.2 Burn injury days: Day 1/Day 3
9.1.2.3 TCI-days: Day 2/Day 4
9.2 Clinical assessments (TME-participants and BI-participants)
9.2.1 Psychometric evaluation
9.2.2 Evaluation of opioid withdrawal (BI-participants Day 0 not included)

9.2.3 Reaction time
9.2.4 Pain assessments
9.2.4.1 TME-participants
9.2.4.2 BI-participants
9.2.5 Secondary hyperalgesia/allodynia
9.2.5.1 TME-participants
9.2.5.2 BI-participants
9.2.6 Pressure pain threshold (TME-participants)
9.2.7 Pin-prick pain threshold (BI-participants)
10. STUDY DRUG AND PHARMACY PROCEDURE 25
10.1 Study drugs
10.1.1Naloxone
10.1.2 Placebo
10.1.3 Rescue drugs
10.2 Blinding procedure, packing and labeling
10.2.1 TME-participants and BI-participants
10.2.2 Manufacturing, packaging and labelling
11. DRUG ADMINISTRATION 26
11.1 Target-controlled-infusion (all participants)
11.2 Infusion pump
11.2.1 TME-participants
11.2.2 BI-participants
12. SIDE EFFECTS 27
12.1 Side effects/Adverse events (AEs)
12.2 Grading of adverse events
12.3 Reporting of AEs and SAEs
12.4 Procedures in case of emergency
13. STATISTICAL ANALYSES 29
13.1 Sample size calculation
13.1.1 TME-participants
13.1.2 BI-participants
13.2 Data processing
13.2.1 TME-participants
13.3.2 BI-participants
14. DATA REGISTRATION 31
14.1 Data registration and control of investigation procedures
14.2 Case Report Form
14.3 GCP-Monitoring
15. MISCELLANEOUS 32
15.1 Insurance
15.2 Financial issues
15.3 Publication of results

SUPPLEMENTAL FILE 1 33
Clinical Opiate Withdrawal Scale (COWS)
SUPPLEMENTAL FILE 2 35
Pain Catastrophizing Scale (PCS)
SUPPLEMENTAL FILE 3 36
Hospital Anxiety and Depression Scale (HADS)

Name and contact information of the persons involved in the study

- **Sponsor and Principal Investigator:**
  Mads U. Werner
  Associate professor (LUND), M.D., Ph.D., D.M.Sc.
  Multidisciplinary Pain Center 7612, Neuroscience Center
  Rigshospitalet and Copenhagen University Hospital
  Blegdamsvej 9
  2100 Copenhagen, Denmark
  Tlf: +45 2825 7703
  Email: mads.u.werner@gmail.com

- **Associate Researcher:**
  Anne Willum
  Research Nurse, M.Sc.
  Multidisciplinary Pain Center 7612, Neuroscience Center
  Rigshospitalet and Copenhagen University Hospital
  Blegdamsvej 9
  2100 Copenhagen, Denmark
  Tlf: +45 2388 0110
  Email: anne.willum@hotmail.com

- **Assistant Researcher:**
  Anders Springborg
  Medical Student
  Multidisciplinary Pain Center 7612, Neuroscience Center
  Rigshospitalet and Copenhagen University Hospital
  Blegdamsvej 9
  2100 Copenhagen, Denmark
  Tlf: +45 3545
  Email: andersspringborg@gmail.com

  Bradley K. Taylor, M.Sc.
  Professor, Head of Department
  Department of Physiology
  MS-508 University of Kentucky Medical Center
  800 Rose Street Lexington, KY 40536-0298, USA
  Email: brad.taylor@uky.edu

  Ola Dale, M.D., D.M.Sc.
  Professor
  Department of Circulation and Medical Imaging
  Norwegian University of Science and Technology
  Prinsesse Kristinas gate 3
  Trondheim
  E-mail: ola.dale@ntnu.no

- **Monitor:**
  Copenhagen University Hospital GCP-unit
  Bispebjerg Hospital, Building 51, 3.sal Bispebjerg Bakke, 23
  2400 København NV, Denmark
  Tlf: +45 35 31 38 90

Sponsor and principal investigator hereby certify that the terms of the Protocol will be followed and that the study will be performed according to the rules of Good Clinical Practice.

Sponsor's and Principal Investigator's name and address:

Mads U. Werner

Associate professor (LUND), M.D., Ph.D., D.M.Sc.
Multidisciplinary Pain Center 7612, Neuroscience Center
Rigshospitalet and Copenhagen University Hospital
Blegdamsvej 9
2100 Copenhagen, Denmark
Tlf: +45 2825 7703
Email: mads.u.werner@gmail.com

Sponsor's and Principal Investigator's date and signature:

January 13th 2016

1. INTRODUCTION AND RATIONALE OF THE STUDY

1.1 Background

Naloxone is a mu-opioid-receptor (MOR) antagonist drug, which dose-dependently exhibits hypo-algesic and hyper-algesic properties [1]. Naloxone (and other MOR-antagonists) have been used in research to study the role of endogenous opioids on central processing of pain. It has been hypothesized that the endogenous opioid modulation of pain is impaired or altered in chronic pain conditions [2–5].
In a previous study using an electrical pain model on human patients, naloxone (21 microg/kg) increased the established area of secondary hyperalgesia [6]. Further, administration of naloxone and naltrexone to animals following resolution of an inflammatory condition, have demonstrated a reinstatement of hypersensitivity to noxious stimulus, indicating latent sensitization [7–9]. It has thus been speculated that endogenous opioids may play an important role in the transition of acute to chronic pain in humans [7,8,10].
Recently, we were unable to show reinstatement of secondary hyperalgesia after resolution of a burn-injury by administrating naloxone in a low dose (21 microg/kg) (H-2-2012-036) [11]. Based on these finding we hypothesized, that the negative results may be due to the low dose of naloxone or insufficient tissue injury to generate latent sensitization.
The systemic doses of opioid antagonists used in animal studies to demonstrate latent sensitization have been 0.3 to 3.0 mg/kg of naltrexone [7] or 3-10 mg/kg of naloxone [12]. Further, high doses of 1-2 mg/kg of naloxone have been used in clinical and experimental psychiatric, endocrinological, neurological and nutritional studies in patients [13–18] and in healthy individuals [19–23]. In one pain related study, 6 mg/kg of naloxone was given to healthy patients intramuscularly [24]. Only mild to moderate, transitory side-effects were recorded in these studies.
We therefore initiated another translational study in which we hypothesized that a higher dose of naloxone (2 mg/kg) would reinstate secondary hyperalgesia in human patients following resolution of a mild burn-injury and thus show latent sensitization in humans. We demonstrated in 4 out of 12 patients, that naloxone administered 7 days after a mild burn injury was associated with reinstatement of secondary hyperalgesia [12]. This study thus raises two important questions: *first*, is it possible to foresee individuals who will demonstrate latent sensitization upon challenge with an injury, and, *second*, will challenge with a larger tissue injury, including more extensive inflammatory reactions, induce a higher relative number of responders.
Consequently, our aim in the present study, is twofold. *First*, to investigate if naloxone will reinstate clinical pain and hyperalgesia after resolution of a more pronounced inflammatory injury in a postsurgical model, i.e., three to four weeks after a unilateral, impacted mandibular third molar extraction procedure. At this time point, patients are expected to be pain free at rest and only have mild pain during specific movements. We expect in this way to contribute with further knowledge on the role of the endogenous opioid-system in latent sensitization, following a mild thermal injury and clinical relevant surgery-related tissue injury. *Second*, in volunteers, to investigate if high-sensitizers (*a priori* developing large areas of secondary hyperalgesia after exposure to a mild burn-injury) are more prone

to demonstrate latent sensitization following naloxone administration, than low-sensitizers (*a priori* developing small areas of secondary hyperalgesia).

The post-surgical pain model, used in the present study, induces deep tissue inflammation and is associated with an increased degree of nociception compared to the superficial burn-injury model, used in our previous studies (H-2-2012-036, H-2-2012-174). In the animal studies on latent sensitization, deep tissue inflammation was induced by plantar incision [7] or injection of complete Freud's adjuvant (CFA) [9]. A pain model, which induces moderate deep tissue inflammation, like post-groin hernia repair (GHR; ongoing study) or post-impacted mandibular third molar extraction (TME; present study), might thus be necessary to generate latent sensitization in humans. The GHR-procedure involves surgical injury to densely innervated soft tissues, and postoperative pain likely, in addition to the inflammatory component, also includes a neuropathic component. Since, the TME-procedure involves damage to the mucoperiosteal tissues, it is of importance to detect if latent sensitization is operative in a predominantly inflammatory pain model [25], compared to the GHR-procedure, a mixed neuropathic and inflammatory pain model. This distinction seems relevant since chronic pain states, from a pathophysiological perspective, are separated into neuropathic, inflammatory or mixed conditions.

In regard to the risk of development of a sustained pain state after the naloxone administration in individuals after TME, we consider it highly unlikely. *First*, naloxone does not affect the production of endogenous opioids, but only acts as a short-acting reversible antagonist of opioids. *Second*, in adults the distribution half-life ($T½\alpha$) of naloxone is 40 to 70 seconds [26], and the elimination ($T½\beta$) half-life, 54 to 64 min [26] [27]. Naloxone has not any known long-acting metabolites and thus there are no pharmacokinetic reasons for a prolonged reversal of endogenous opioids. *Third*, in studies with rodents, administration of naltrexone or naloxone has caused transient episodes of hypersensitivity with duration of 60 to 90 min [9]. Repeated administration of naltrexone, over the course of months, has confirmed full reversibility of the hypersensitivity episodes. *Fourth*, in our recent patient-study with high dose naloxone (H-2-2012-174) in 4 of 12 individuals responding to naloxone, very short lasting changes in hypersensitivity were seen [12]. Thus it is our strong belief that the likelihood of development of significant or serious adverse effects, including sustained pain, is minimal in this study.

1.2 References

1. Brennum J, Kaiser F, Dahl JB (2001) Effect of naloxone on primary and secondary hyperalgesia induced by the human burn injury model. Acta Anaesthesiol Scand 45: 954-960. aas450806 [pii].
2. Pielsticker A, Haag G, Zaudig M, Lautenbacher S (2005) Impairment of pain inhibition in chronic tension-type headache. Pain 118: 215-223. S0304-3959(05)00418-5 [pii];10.1016/j.pain.2005.08.019 [doi].
3. Price DD, Staud R, Robinson ME, Mauderli AP, Cannon R, Vierck CJ (2002) Enhanced temporal summation of second pain and its central modulation in fibromyalgia patients. Pain 99: 49-59. S0304395902000532 [pii].
4. Staud R, Weyl EE, Price DD, Robinson ME (2012) Mechanical and heat hyperalgesia highly predict clinical pain intensity in patients with chronic musculoskeletal pain syndromes. J Pain 13: 725-735. S1526-5900(12)00602-5 [pii];10.1016/j.jpain.2012.04.006 [doi].
5. van Wilgen CP, Keizer D (2012) The sensitization model to explain how chronic pain exists without tissue damage. Pain Manag Nurs 13: 60-65. S1524-9042(10)00032-9 [pii];10.1016/j.pmn.2010.03.001 [doi].

6. Koppert W, Filitz J, Troster A, Ihmsen H, Angst M, Flor H, Schuttler J, Schmelz M (2005) Activation of naloxone-sensitive and -insensitive inhibitory systems in a human pain model. J Pain 6: 757-764.
7. Campillo A, Cabanero D, Romero A, Garcia-Nogales P, Puig MM (2011) Delayed postoperative latent pain sensitization revealed by the systemic administration of opioid antagonists in mice. Eur J Pharmacol 657: 89-96. S0014-2999(11)00117-8 [pii];10.1016/j.ejphar.2011.01.059 [doi].
8. Taylor BK, Corder G (2014) Endogenous analgesia, dependence, and latent pain sensitization. Curr Top Behav Neurosci 20: 283-325. 10.1007/7854_2014_351 [doi].
9. Corder G, Doolen S, Donahue RR, Winter MK, Jutras BL, He Y, Hu X, Wieskopf JS, Mogil JS, Storm DR, Wang ZJ, McCarson KE, Taylor BK (2013) Constitutive mu-opioid receptor activity leads to long-term endogenous analgesia and dependence. Science 341: 1394-1399. 341/6152/1394 [pii];10.1126/science.1239403 [doi].
10. Rivat C, Laboureyras E, Laulin JP, Le RC, Richebe P, Simonnet G (2007) Non-nociceptive environmental stress induces hyperalgesia, not analgesia, in pain and opioid-experienced rats. Neuropsychopharmacology 32: 2217-2228. 1301340 [pii];10.1038/sj.npp.1301340 [doi].
11. Pereira MP, Werner MU, Ringsted TK, Rowbotham MC, Taylor BK, Dahl JB (2013) Does naloxone reinstate secondary hyperalgesia in humans after resolution of a burn injury? A placebo-controlled, double-blind, randomized, cross-over study. PLoS One 8: e64608. 10.1371/journal.pone.0064608 [doi];PONE-D-13-08611 [pii].
12. Pereira MP, Donahue RR, Dahl JB, Werner M, Taylor BK, Werner MU (2015) Endogenous Opioid-Masked Latent Pain Sensitization: Studies from Mouse to Human. PLoS One 10: e0134441. 10.1371/journal.pone.0134441 [doi];PONE-D-14-43604 [pii].
13. Cohen MR, Pickar D, Cohen RM (1985) High-dose naloxone administration in chronic schizophrenia. Biol Psychiatry 20: 573-575. 0006-3223(85)90030-7 [pii].
14. Cohen MR, Cohen RM, Pickar D, Sunderland T, Mueller EA, III, Murphy DL (1984) High dose naloxone in depression. Biol Psychiatry 19: 825-832.
15. Adams HP, Jr., Olinger CP, Barsan WG, Butler MJ, Graff-Radford NR, Brott TG, Biller J, Damasio H, Tomsick T, Goldberg M, . (1986) A dose-escalation study of large doses of naloxone for treatment of patients with acute cerebral ischemia. Stroke 17: 404-409.
16. Barsan WG, Olinger CP, Adams HP, Jr., Brott TG, Eberle R, Biller J, Biros M, Marler J (1989) Use of high dose naloxone in acute stroke: possible side-effects. Crit Care Med 17: 762-767.
17. Olinger CP, Adams HP, Jr., Brott TG, Biller J, Barsan WG, Toffol GJ, Eberle RW, Marler JR (1990) High-dose intravenous naloxone for the treatment of acute ischemic stroke. Stroke 21: 721-725.
18. Federico F, Lucivero V, Lamberti P, Fiore A, Conte C (1991) A double blind randomized pilot trial of naloxone in the treatment of acute ischemic stroke. Ital J Neurol Sci 12: 557-563.
19. Cohen MR, Cohen RM, Pickar D, Kreger D, McLellan C, Murphy DL (1985) Hormonal effects of high dose naloxone in humans. Neuropeptides 6: 373-380.
20. Cohen MR, Cohen RM, Pickar D, Weingartner H, Murphy DL (1983) High-dose naloxone infusions in normals. Dose-dependent behavioral, hormonal, and physiological responses. Arch Gen Psychiatry 40: 613-619.
21. Cohen MR, Cohen RM, Pickar D, Murphy DL, Bunney WE, Jr. (1982) Physiological effects of high dose naloxone administration to normal adults. Life Sci 30: 2025-2031.
22. Cohen MR, Cohen RM, Pickar D, Murphy DL (1985) Naloxone reduces food intake in humans. Psychosom Med 47: 132-138.
23. Cohen MR, Cohen RM, Pickar D, Weingartner H, Murphy DL, Bunney WE, Jr. (1981) Behavioural effects after high dose naloxone administration to normal volunteers. Lancet 2: 1110.
24. Edwards RR, Ness TJ, Fillingim RB (2004) Endogenous opioids, blood pressure, and diffuse noxious inhibitory controls: a preliminary study. Percept Mot Skills 99: 679-687.
25. Singla NK, Desjardins PJ, Chang PD (2014) A comparison of the clinical and experimental characteristics of four acute surgical pain models: Dental extraction, bunionectomy, joint replacement, and soft tissue surgery. Pain 155: 441-456. S0304-3959(13)00495-8 [pii];10.1016/j.pain.2013.09.002 [doi].
26. Glass PS, Jhaveri RM, Smith LR (1994) Comparison of potency and duration of action of nalmefene and naloxone. Anesth Analg 78: 536-541.
27. Chamberlain JM, Klein BL (1994) A comprehensive review of naloxone for the emergency physician. Am J Emerg Med 12: 650-660.

2. AIM

The principal aims of the study are:

***** *first*, to investigate whether the administration of naloxone, a selective mu-opioid receptor (MOR) antagonist, can re-introduce clinical pain (at rest, during movement and in pressure-evoked condition) and hyperalgesia three to four weeks after a unilateral, mandibular third molar extraction procedure (TME).

***** *second*, to assess predictability of the latent sensitization response, assessed as naloxone-induced reinstatement of secondary hyperalgesia after resolution of an experimental burn-injury, according to individual sensitization phenotype (high-sensitizers *vs.* low-sensitizers).

3. OUTCOMES

3.1 Primary Outcome

3.1.1 Impacted mandibular third molar extraction (TME) study

- A composite measure of pain (numerical rating scale [NRS]; 0 = no pain; 10 = worst perceivable pain) during rest + masticatory pain + pain during external algometry (100 kPa) at the injury site, assessed 4 weeks (± 3 days) after uncomplicated extraction of an impacted mandibular third molar.

3.1.2 Burn injury (BI) study

- Areas of secondary hyperalgesia assessed by a weighted-pin instrument (256 mN) 0, 1, 2 and 165-169 hrs (during TCI [time; 10-15"[A]; 44-49"; and 70-75"]) after the BI.

3.2 Secondary Endpoints

3.2.1 Impacted mandibular third molar extraction (TME) study

- Secondary hyperalgesia/allodynia area at mandibular skin sites directly overlying surgical and contralateral side (nylon monofilament (nominal value 4.93 [bending force: mean + SD = 69 ± 14 mN])
- Online Reaction Time (http://getyourwebsiteherecom/jswb/rttest01.html)
- Hospital Anxiety and Depression Scale (HADS; only pre-infusion)
- Pain Catastrophizing Scale (PCS; only pre-infusion)
- Clinical Opiate Withdrawal Scale (COWS; cf. p. 31 to 32)

3.2.2 Burn injury (BI) study

- Pain during BI (NRS)
- Pin-prick pain thresholds assessed (PPT) by weighted-pin instruments at primary and secondary hyperalgesia areas (0, 1, 2, 165 and 165-169 hrs (during TCI [time; 15-25"; 44-49"; and 70-75"]) after BI
- Online Reaction Time (http://getyourwebsiteherecom/jswb/rttest01.html)

[A] The double prime characters (") indicate min.

- Hospital Anxiety and Depression Scale (HADS; only pre-BI)
- Pain Catastrophizing Scale (PCS; only pre-BI)
- Clinical Opiate Withdrawal Scale (COWS; cf. p. 31 to 32)

4. Ethical considerations

4.1 General ethical considerations

The study will be conducted in accordance to the principles of the Helsinki Declaration with updated amendments.[B] The protocol will be submitted for approval to the Regional Committee of Research Ethics, Danish Health and Medicines Authority and the Danish Data Protection Agency. Investigators will also report significant or major changes to the protocol to these agencies. The study will be registered in EUDRACT and the international database www.clinicaltrial.gov.

Comparable studies have previously been approved of the Regional Committee of Research Ethics of the Capital Region (H-2-2012-174; H-15002712), the Danish Health and Medicines Agency (2012-005663-17; 2015-000793-36) and the Danish Data Protection Agency (2013-41-1475; RH-2014-30-1150, I-Suite nr.: 02815). These studies were also registered in ClinicalTrials.gov (NCT01992146) and EudraCT (2012-005663-27 DK 20130222; 2015-000793-36 DK 20150216; 2015-000815-42 DK 20150216).

4.2 Risks and inconveniences for the study subjects

4.2.1 Study drug (all participants)

4.2.1.1 Acute effects

Since the study drug will be administered intravenously, a peripheral intravenous catheter will be placed. This may cause discomfort related to skin puncture by the needle. When the venous catheter is removed a small hematoma may develop that, however, will disappear by itself within a few days.

In previous studies, naloxone given in doses of 2-4 mg/kg i.v. only caused mild side-effects in a minority of volunteers or patients (30%). These side-effects include: nausea, vomiting, weakness, fatigue, tremor, elevations of blood pressure and respiratory rate. Interestingly, in a placebo-controlled, randomized crossover experimental pain study, naloxone 6 mg/kg i.m. was administered to 6 healthy patients. The subjects were not able to discriminate between the subjective effects of naloxone and placebo (!)[23].

In a recent study (H-2-2012-174) we administered naloxone 2.0 mg/kg to 15 healthy subjects. Six study subjects reported mild side effects: 3 subjects experienced mild to moderate tiredness, 2 subjects experienced a frontal headache and 1 subject experienced epigastric pain. In addition, 1 subject reported intense photophobia, which lasted until the following day. During infusion of the drug, participants will be monitored with ECG and measurements of pulse oximetry, blood pressure and respiratory rate. When naloxone is given, a physician and a nurse will be present and attend the patient or volunteer if potential side effects should require any kind of treatment.

By administration of naloxone to TME-participants that have recently undergone surgery, there is a

[B] WMA Declaration of Helsinki - Ethical Principles for Medical Research Involving Human Subjects (http://www.wma.net/en/30publications/10policies/b3/ Accessed 12/07/2014)

possibility that participants will experience short lasting pain or unpleasantness from the surgical area. This may be uncomfortable for the participant, but is essential to answer the questions asked in this study. If requested by the participant, the rapid-onset, analgesic, rescue-opioid, alfentanil, will be administered, which in less than one min completely will antagonize the effects of naloxone. Alfentanil is a well-known drug used in anesthesia for immediate relief of acute pain. Alfentanil is initially given 7-15 mikrog/kg (1-2 ml/70 kg) and is administered in a titrated fashion until the desired analgesic effect is achieved. Common dose-dependent side effects are nausea, vomiting and sedation (> 10%). During the naloxone-infusion the participant's resting pain is monitored. The naloxone-infusion is immediately stopped if the participant experiences resting pain levels ≥ 5 (NRS 0-10).

4.2.1.2 Protracted effects

In regard to the risk of development of a sustained pain state after the naloxone administration in individuals after impacted mandibular third molar extraction, we consider it highly unlikely (cf. page 5).

4.2.1.3 Conclusion

As the pain will dissolve rapidly spontaneously, or if necessary, after antagonizing and abolishing the effects of the study drug with alfentanil, the participant will be well-informed about the possibility that this can occur. Thus we believe there will not be significant ethical issues in this study.

The study is considered by the research group to be important, since the result will enhance our understanding of the transition from acute to persistent pain states, and thereby, may improve stratification and management strategies.

4.2.2 Burn injury (BI-particiants)

4.2.2.1 Adverse effects

The first degree burn injury is a moderately painful procedure inducing signs of a slight sunburn with erythema, hyperemia, hyperalgesia and hypersensitivity. These signs subside in most cases 24 to 48 hrs, without leaving residual signs. However in 1-2% of participants hyperpigmentation is evident.

If the participant experiences severe pain during induction of the burn injury the participant is able to discontinue the heating by pushing a button terminating the stimulus or by saying so to the investigator. In case of a malfunctioning, overheating contact thermode a more severe second to third degree burn injury has been described. Due to the rigorous, compulsory testing paradigms of our thermodes, we have not experienced any thermal adverse effects during the last five years with the BI-model (H-2-2010-115; H-2-2012-036; H-2-2012-174; H-1-2013-045; H-4-2013-013), including more than 350 burn injuries.

4.2.2.2 Conclusion

The investigators do not believe that there will be any significant ethical issues using the burn injury in this study.

4.3 Informed Consent

4.3.1 Impacted mandibular third molar extraction study (TME-participants)

All included TME-participants have been submitted to unilateral, primary, uncomplicated, impacted mandibular third molar extraction surgery at Dept. Oral Surgery (att. senior clinical instructor Lars Pallesen DDS), School of Dentistry, University of Copenhagen (section 29.1.14; Nørre Allé 20; 2300 Copenhagen O). The TME-participants will receive the written information about the study by the investigator before the operation. The investigator will give verbal information in a quiet room and inform the participant that he has the right to bring a relative for the information, and to have time to consider participation in the study. Two weeks (± 3 days) after the surgery the investigator will make a telephone call, four weeks [± 3 days] after the TME-surgery, and make an appointment with the subject for the examination days (Day 1; Day 2).

Day 1 the investigator will repeat the oral information and will answer any questions the TME-participants may have. The written consent will be obtained on Day 1 of the study before any assessments will be done. If the participant needs more time before signing the informed consent a new Day 1 will be scheduled. The participant will be informed that the participation in the study is voluntary and that they may withdraw from the study at any time without further explanation. This will not have any consequence for ongoing or future treatments at the involved departments or hospitals. In addition, the participant will be informed that the investigator may exclude them from the study at any time. TME-participants will receive an hourly payment for attending the study of 150 DKR (25 USD) and if required by the participant, reimbursement for transportation costs.

4.3.2 Burn injury study (BI-participants)

The BI-participants will be recruited from our registry of volunteers who previously have participated in experimental pain studies at the Multidisciplinary Pain Center 7612, Rigshospitalet, and, specifically, have given their verbal or written consent, that they may be contacted in future research. In addition, if the investigators experience difficulty in recruitment of participants, an advertisement will be placed in a Danish E-site for recruitment of volunteers in medical research (forsoegsperson.dk).

The BI-participants will receive the written information about the study by the investigator. The investigator will schedule a physical meeting with the BI-participant and inform the participant that he has the right to bring a relative or a friend for the information meeting. At the meeting, that takes place in a quiet room, in an undisturbed environment, the investigator will then render verbal information concerning the study and answer the BI-participant's and the relative's questions. The BI-participant is informed that he has time to consider participation in the study. Two days after the meeting the investigator will call or send an E-mail, making an appointment with the BI-participant for the study day.

At the study day the investigator will repeat the verbal information and will answer any questions the BI-participants may have. The written consent will be obtained on the study day before any assessments will be done. If the BI-participant needs more time before signing the informed consent a new study day will be scheduled. The participant will be informed that the participation in the study is voluntary and

that he may withdraw from the study at any time without further notice or explanation. This will not have any consequence for ongoing or future treatments at the involved departments or hospitals. In addition, the participant will be informed that the investigator may exclude them from the study at any time. BI-participants will receive an hourly payment for attending the study of 150 DKR (25 USD) and if required by the BI-participant, reimbursement for transportation costs.

4.4 Data Registration and Protection

The trial will be reported to the Data Protection Agency. The "Act on Processing of Personal Data" will be respected. All the participants personal data will be kept confidential. All information and the reporting of individual test-results will be treated confidentially. The participant's data will be anonymous and the persons involved in the study will adhere to the secrecy legislation.

Data collected in the form of Case Report Form, signed informed consent forms and records created for the study will only be made available for monitoring and inspection by authorized representatives of relevant authorities, including the Good Clinical Practice (GCP) unit of the Copenhagen University Hospital, the Regional Committee of Research Ethics, the Danish Health and Medicines Authority and the Danish Data Protection Agency. The study will follow the guide-lines and rules of GCP and Good Medical Practice (GMP).

5. Timeline and Location of the Study

5.1 Estimated time plan (all participants)

Study start:	**February 1st 2016**	**01.02.2016**
Study completion:	**March 31st 2017**	**31.03.2017**

5.2 Duration of examination days

5.2.1 TME-participants

The two examination days are identical in set-up and will require total 6 hrs of participation.

5.2.2 BI-participants

The five examination (Days 0-4) days require a total 17½ hrs of participation. Day 0 is a screening day (1½ hrs) selecting high- and low-sensitizers eligible for examination at Days 1-4. Day 1 and Day 3 are BI-days (5 hrs), and, Day 2 and Day 4 are TCI-days (11 hrs).

5.3 Location (all participants)

Departments of Neuroanaesthesia 3042 and Multidisciplinary Pain Center 7612, both at Neuroscience Center, Rigshospitalet. The study takes place in a quiet room (22-25 ºC, relative humidity [RH] 20-75%).

6. Study Design

6.1 TME-participants

Study type:	Randomized, placebo-controlled, double-blind crossover
Randomization:	Computer generated randomization list, closed envelopes
Phase:	Phase 2
Blinding:	Double-blind, crossover
Subjects:	Participants with unilateral, primary, uncomplicated, impacted mandibular third molar extraction
Number:	14 evaluable subjects (maximum included number: 18)

6.2 BI-participants

Study type:	Randomized, placebo-controlled, double-blind crossover
Randomization:	Computer generated randomization list, closed envelopes
Phase:	Phase 2
Blinding:	Double-blind, crossover
Subjects:	Healthy volunteers
Number:	Day 0: 80 subjects; Day 1-4: 40 evaluable subjects

7. SELECTION CRITERIA

7.1 Inclusion Criteria

7.1.1 TME-participants

TME-participants must meet all the following criteria to be eligible to enroll in the study:

- Healthy male
- Age above 18 yrs and below 65 yrs
- Signed informed consent
- Participants submitted to unilateral, primary, impacted, uncomplicated mandibular third molar extraction 4 weeks (± 3 days) prior to examination Day 1.
- Standardized surgical procedure.
- Urin-sample *without* traces of opioids (morphine, methadon, buprenorphine, codeine, tramadol, ketobemidone, oxycodone, hydromorphone, dextromethorphan)
- ASA I-II
- Body mass index (BMI): 18 < BMI < 30 kg/m^2

7.1.2 BI-participants

BI-participants must meet all the following criteria to be eligible to enroll in the study Day 0:

- Healthy male
- Age above 18 yrs and below 35 yrs
- Signed informed consent
- Urin-sample *without* traces of opioids (morphine, methadon, buprenorphine, codeine, tramadol, ketobemidone, oxycodone, hydromorphone, dextro-methorphan)
- ASA I

- Body mass index (BMI): 18 < BMI < 30 kg/m^2

In addition, BI-participants must meet the following criteria to be eligible to further enroll in the study Day 1-4:

- Secondary hyperalgesia areas[C] 1 hr after a burn injury (47C°, 2.5x5.0 cm^2, 7") either $\geq$ 37 cm^2 (high-sensitizers) or $\leq$ 22 cm^2 (low-sensitizers). The selection into high-sensitizers and low-sensitizers is made during a separate test day (Day 0).

7.2 Exclusion criteria

7.2.1 TME-participants

TME-participants, who meet one or more of the following criteria, are not suitable for inclusion in this study:

- Participants, who do not speak or understand Danish
- Participants, who cannot cooperate with the investigation
- Participants, who have had previous surgery in the mandibular region
- Participants with pain at rest > 3 (NRS [0: no pain; 10: worst perceivable pain])
- Activity-related pain in the surgical field > 5 (NRS)
- Allergic reaction against morphine or other opioids (including naloxone),
- Abuse of alcohol or drugs – according to investigator's evaluation
- Use of psychotropic drugs (exception of SSRI)
- Neurologic or psychiatric disease
- Chronic pain condition
- Regular use of analgesic drugs
- Skin lesions or tattoos in the assessment areas
- Nerve lesions in the assessment sites (e.g., after trauma, dental surgery)
- Use of prescription drugs one week before the trial
- Use of over-the-counter (OTC) drugs 48 hours before the trial

7.2.2 BI-participants

BI-participants, who meet one or more of the following criteria, are not suitable for inclusion in this study:

- Participants, who do not speak or understand Danish
- Participants, who cannot cooperate with the investigation
- Allergic reaction against morphine or other opioids (including naloxone)
- Abuse of alcohol or drugs – according to investigator's evaluation
- Use of psychotropic drugs (exception of SSRI)
- Neurologic or psychiatric disease
- Signs of neuropathy in the examination region
- Previous severe trauma to the lower legs with sequelae
- Scarring or tattoos in the examination areas
- Chronic pain condition
- Regular use of analgesic drugs
- Use of prescription drugs one week before the trial

[C] Secondary hyperalgesia areas are calculated after subtracting the primary hyperalgesia area (12.5 cm^2). Data from: Ringsted TK, Enghuus C, Petersen MA, Werner MU (2015) Demarcation of secondary hyperalgesia zones: Punctate stimulation pressure matters. J Neurosci Methods 256: 74-81.

- Use of over-the-counter (OTC) drugs 48 hours before the trial
- Does not develop measurable secondary hyperalgesia areas after BI

8. Practical considerations

8.1 Economical issues

Mads U. Werner has in collaboration with the research partners (Anne Willum, Bradley Taylor, Manuel P. Pereira, Jørgen B. Dahl, and Mads Kreilgaard) taken the initiative to this study. Expenses of the study include costs regarding: reimbursement to the participants, and, costs of the study drug, submission fees to the Danish Medicines Agency, salary to research nurse and miscellaneous (e.g. statistical assistance, computer-hardware and computer-software). All staff members involved in the study are employed at the Multidisciplinary Pain Center 7612, Neuroscience Center, Rigshospitalet. Incurred costs will be paid from the research fund of Mads U. Werner, subjected to audit from the hospital-administration. In a consortium agreement with the University of Kentucky Research Foundation we have September 2015 received a research award from the National Institute of Health (NIH), U.S.A. for research entitled, "Long-term activation of spinal opioid analgesia after inflammation". Major part of the cost will be paid from this resource.. A budget specification is given in paragraph 15.2 (page 33).

Neither the sponsor/principal investigator, nor any of the research-partners have any financial interest in the study.

8.2 Participant inclusion and withdrawal from the study

- A participant, who has completed the trial, is a participant, who has followed the complete treatment and assessment plan of the study in the required 2 hours of both study days.
- A participant, who has not completed the trial, is a participant, who was included in the trial (i.e. has signed the informed consent), but did not complete the trial, regardless of having received the study drug or not.
- If a participant has not completed the trial, the procedures regarding this participant must be specified – this is also valid for drop-outs – and also what data is collected from these subjects.

8.3 Reasons to withdraw a participant from the study

A participant can be excluded from the study under the following conditions:

- If the participant wishes to withdraw from the trial
- Violation of the protocol rules

8.4 Procedures for participants withdrawing from the study

In accordance with the Declaration of Helsinki with appropriate amendments, the participants have the right to end their participation in the study at **any** time for **any** reason. The investigator has also the right to withdraw a participant from the study at **any** time. Reasons for a participant withdrawing before the end of the study will be recorded and documented in the participant's Case Report Form.

9. METHODS

9.1 Study algorithms

9.1.1 TME-study

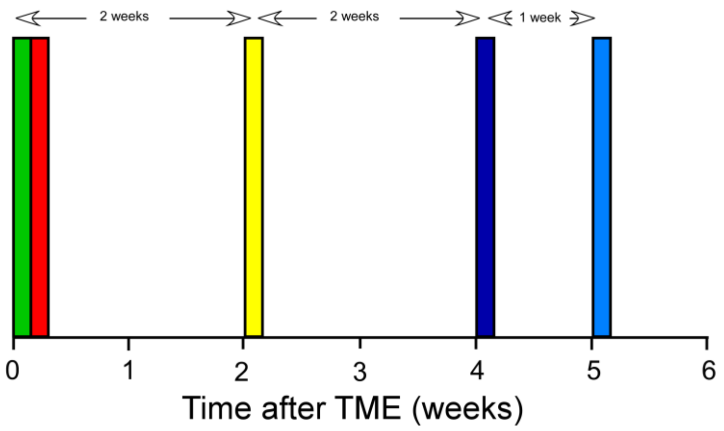

Fig 1. Study layout for impacted third molar extraction (TME) participants. The general timelines of the impacted third molar extraction (TME) study. Information day (green bar) where participants are informed about the study. The day of surgery (red), the follow-up phone call (yellow; 2 weeks [+ 3 days] after TME), the first examination day (Day 1; dark blue; 4 weeks [+ 3 days]) and the second examination day (Day 2; blue; exactly 7 days after Day 1).

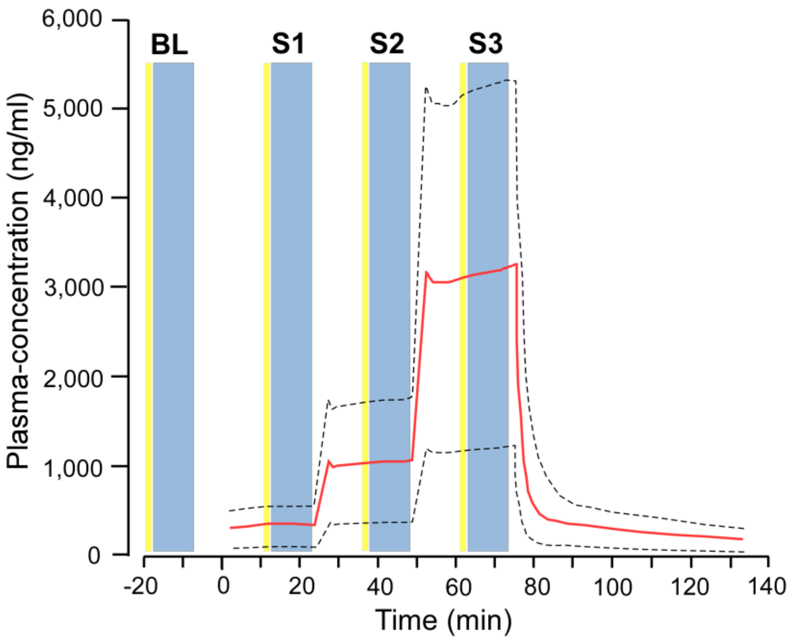

Fig 2. Test-algorithm Day 1/2 for TME-participants and Day 2/4 for BI-participants with superimposed naloxone plasma-concentration curves. Median plasma-concentration (red) with 95% CI (dashed black lines) during a three-step target-controlled-infusion (TCI). Naloxone is given at step one with 0.25 mg/kg, step two 0.75 mg/kg and step three 2.25 mg/kg. Yellow timeline columns represent ratings with Clinical Opiate Withdrawal Scale (COWS) and blue columns indicate sensory testing (pain during rest, movement and 100 kPa pressure; pressure algometry; secondary hyperalgesia area). **BL** = baseline assessments (-20" to -8"; please, observe for BI-participants baseline assessments are made – 2hrs 20"

to – 2hrs 8'' before start of TCI); **S1** = step one assessments (15'' to 25''); **S2** = step two assessments (39'' to 49''); **S3** = step three assessments (65'' to 75'').

At the follow-up phone call 2 weeks (+ 3 days) after surgery any questions the participant may have regarding the study will be discussed. The post-surgical pain intensity and signs of post-surgical complications will be penetrated (swelling, pain, fever, malaise, excessive lymph-node swelling).

The study drug (naloxone or placebo) will be given in a randomized fashion on the examination days, Day 1 and Day 2. Day 1 takes place 4 weeks (+ 3 days) after surgery. Day 1 and Day 2, separated by exactly 7 days, are at Department of Neuroanaesthesia 3042, Rigshospitalet (Fig. 1). Procedures on Day 1 and Day 2 will be performed by a physician and a research-nurse.

The agenda for the examination days are:

9.1.2.1.1 Day 1/Day 2

- Assessment of inclusion and exclusion criteria
- The participant gives his informed consent (only Day 1)
- The participant fills out the Hospital Anxiety and Depression Scale (HADS; only Day 1)
- The participant fills out the Pain Catastrophizing Scale (PCS; only Day 1)
- The examiner fills out the Clinical Opiate Withdrawal Scale (COWS)
- Brief medical examination (only Day 1):
    - Medical history
    - Auscultation of heart and lungs
    - Blood pressure and heart rate
    - Routine palpation of lymphatic nodes in the submandibular regions, the supraclavicular fossae and the axillae.
    - Assessment of deep tendon reflexes
- Urine-sample to test for opioids (sample is discarded just after the analysis).
- Randomization (consecutively by pre-specified randomization)
- Monitoring with three-lead ECG and measurements of pulse oximetry, blood pressure and respiratory rate is started.
- Baseline assessments are performed at **time – 20'' to – 8''** (Fig. 2):
    - Reaction time assessed as Online Reaction Time.
    - Pain rating (0-10, NRS) at rest in the supine position.
    - Pain rating (0-10, NRS) during a standardized mastication procedure.
    - Pain rating (0-10, NRS) during pressure-algometry applied perpendicularly at the mandibular skin sites overlying the surgical area and the contralateral mirror-site.
    - Secondary hyperalgesia/allodynia at surgical site and contralateral mirror-site at the mandibular skin sites overlying the surgical area and the contralateral mirror-site.
- **Time 0'' to 25''**: **First** step target-controlled-infusion (TCI; including a bolus dose) of naloxone/placebo infusion (**Fig. 2**)
- **Time 13'' to 15''**: The examiner assesses COWS-rating

- **Time 15'' to 25''**: Identical assessments as made at baseline are repeated
- **Time 25'' to 50''**: **Second** step TCI (including a bolus dose) of naloxone/placebo infusion (**Fig. 2**)
- **Time 37'' to 39''**: The examiner assesses COWS-rating
- **Time 39'' to 49''**: Identical assessments as made at baseline are repeated
- **Time 50'' to 75''**: **Third** step TCI (including a bolus dose) of naloxone/placebo infusion (**Fig. 2**)
- **Time 63'' to 65''**: The examiner assesses COWS-rating
- **Time 65'' to 75''**: Identical assessments as made at baseline are repeated
- **Time 75''**: End of naloxone/placebo infusion
- Monitoring data are continuously registered and documented
- Potential side-effects are continuously registered and documented
- **Time 75'' – 180''**: observation period where the participant is allowed to drink, eat and begin to ambulate. Monitoring is continued until 100"
- **Time 180''**: the participant is discharged

The participants will be contacted by phone (AW) the day after each examination day to check up on potential side effects. The examiner (MUW) will be on call on mobile at the request of the participant for 24 hrs after each examination day.

9.1.2 BI-study

Participants are screened during a separate day (Day 0; 1½ hrs; Fig. 3): a burn injury is induced and secondary hyperalgesia areas are assessed. The boundaries of 25% and 75% quartiles for secondary hyperalgesia areas (22 cm^2 [low-sensitizers] and 37 cm^2 [high-sensitizers], respectively [Ringsted TK,et al. (2015) J Neurosci Methods 256: 74-81]) are used to allocate participants into low-sensitizers (areas $\leq$ 22 cm^2) and high-sensitizers (areas $\geq$ 37 cm^2). Data from the 80 participants are used to determine contemporary and probably more accurate boundaries of 25% and 75% quartiles for secondary hyperalgesia areas! Only low-sensitizers and high-sensitizers continue in the study, the remaining participants are excluded.

Day 1 and Day 3 are identical testing days with induction of BI's and sensory assessments (Figs. 3-4). Day 2 and Day 4 are identical TCI-testing days (Figs. 3-4), with participants randomized and allocated to receive either placebo or naloxone.

9.1.2.1 Screening day: Day 0 (Fig. 3).

- The participants have been instructed to use a hair trimmer in the test areas on both lower legs, two days before each study session.
- Assessment of inclusion and exclusion criteria
- The participant gives his informed consent
- The participant fills out the Hospital Anxiety and Depression Scale (HADS)
- The participant fills out the Pain Catastrophizing Scale (PCS)

- The examiner fills out the Clinical Opiate Withdrawal Scale (COWS)
- Brief medical examination:
  - Medical history
  - Auscultation of heart and lungs
  - Blood pressure and heart rate
  - Routine palpation of lymphatic nodes in the submandibular regions, the supraclavicular fossae and the axillae.
  - Assessment of deep tendon reflexes
- Urine-sample to test for opioids (sample is discarded just after the analysis).
- **Baseline assessments** (all sensory assessments are made at the **left** leg):
  - The corners of the BI-area is outlined by a marker on the medial aspect of the lower leg with the upper margin 11 cm below the medial meniscus and the anterior border 7 cm behind the anterior margin of the tibia.
  - The secondary hyperalgesia area is determined using a “weighted-pin” stimulator (cf. 9.2.5.2). A **green** marker is used to indicate the transition zone.
  - Pin-prick pain thresholds (PPTs) in the primary BI-area and in the secondary hyperalgesia area are assessed by “weighted-pin” stimulators (cf. 9.2.7) .
- **Burn injury induction**:
  - A first-degree BI is induced by a computerized contact thermode (MSA Thermal Stimulator, Somedic AB, Hörby, Sweden; 5.0 x 2.5 cm^2; 47.0ºC, 420 s) applied with gentle pressure at the pre-specified area.
  - Pain intensity is assessed by NRS (0-10) during the BI-induction (cf. 9.2.4.2).
- **Post-injury assessments**:
  - **Time 1 hr 7''** after start of induction, secondary hyperalgesia areas are demarcated (cf. 9.2.5.2), using a **red** marker.
  - PPT’s are assessed, as described above.
  - The demarcations of hyperalgesia areas are transferred to clear acetate sheets.
  - The sheets are scanned, and calculations of the octagon areas (total area minus the area of thermode) are by a computer program (Canvas 12.0, ACD International Systems, Victoria, Canada).
  - The participants are allocated to low-sensitizers and high-sensitizers

9.1.2.2 Burn injury days: Day 1 (right leg)/Day 3 (*left leg*; [Figs. 3 and 4]):

- The participants have been instructed to use a hair trimmer in the test areas on both lower legs, two days before each study session.
- It is verified that the participant belongs to either the low-sensitizer group or the high-sensitizer group.
- Urine-sample to test for opioids (sample is discarded just after the analysis).
- **Baseline assessments**, as previously described (Day 0; Fig. 4).

- **Burn injury induction**, as previously described (Day 0; Fig. 4).
    - **Post-injury assessments**, as previously described (Day 0; Fig. 4). However, an additional 2 hrs post-injury assessment is made at **time 2 hrs 7''** after induction of the burn injury (cf. 9.2.5.2), using a **green** marker.

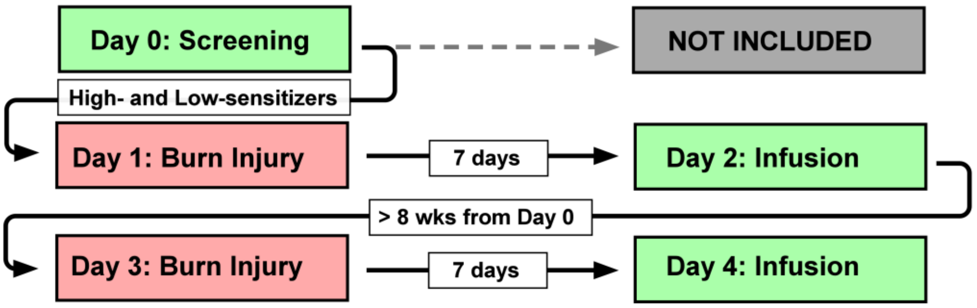

Fig 3. General study layout for the burn injury (BI) study. BIs are induced Day 0, Day 1 and Day 3. Day 0 is a screening day, including high-sensitizers (large secondary hyperalgesia areas; 75% quartile) and low-sensitizers (small secondary hyperalgesia areas 25% quartile). Day 1 and Day 3 include induction of BIs in high- and low-sensitizers. Target-controlled-infusion (TCI) days are Day 2 and Day 4, with randomized allocation between placebo and naloxone. The time line between Day 1 and Day 2, and, Day 3 and Day 4, is 7 days. The timeline between Day 3 and Day 0 is 8 weeks or more.

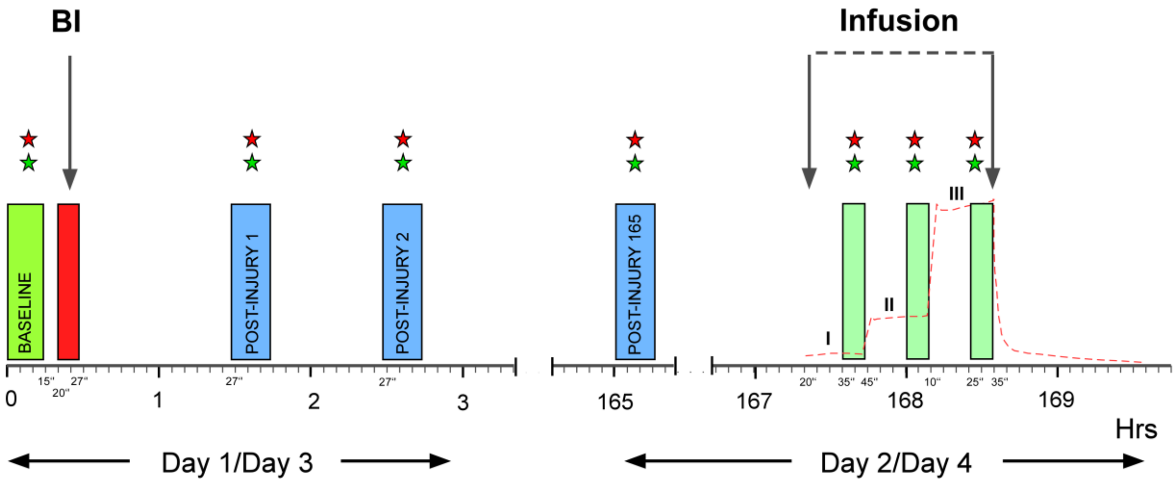

Fig 4. Study layout for the burn injury (BI) study. Day 1/Day 3 includes induction of BI (mild thermal injury; 47C°, 7") with baseline assessments (green rectangle), burn injury (red) and post-injury assessments (blue). Day 2/Day 4 include a pre-drug assessment (blue; post-injury 165 hrs), drug-infusions (naloxone or placebo; 167-168 hrs) and assessments during target-controlled-infusion ([TCI]; light green). The estimated plasma concentrations are superimposed in dashed red line. Roman numerals indicate the three TCI-steps. Stars indicate assessments of pin-prick pain thresholds (red), and secondary hyperalgesia areas (green star).

9.1.2.3 TCI-days: Day 2 (right leg)/Day 4 (*left leg*; [Figs. 3 and 4]):

The study drug, naloxone will be given by a TCI-regimen at the Multidisciplinary Pain Center 7612 or Department of Neuroanaesthesia 3042, both at Rigshospitalet. Procedures on the study day will be performed by a physician and a research-nurse.

- The participants have been instructed to use a hair trimmer in the test areas on both lower legs, two days before each study session.

- The examiner fills out the Clinical Opiate Withdrawal Scale (COWS).
- Urine-sample to test for opioids (sample is discarded just after the analysis).
- Online Reaction Time is measured, as previously described (Day 0).
- Randomization (consecutively by pre-specified randomization)
- Monitoring with three-lead ECG and measurements of pulse oximetry, blood pressure and respiratory rate is started.

- **Time 165 hrs** after induction of the BI Baseline, **Baseline assessments**, as previously described (Day 0), is performed (Fig. 4). A **blue** marker is used to demarcate the secondary hyperalgesia area.
- **Time 167 hrs 20''** to **167 hrs 45''**: **First** step target-controlled-infusion (TCI; including a bolus dose) of naloxone/placebo infusion (Fig. 4).
- **Time 167 hrs 33''** to **167 hrs 35''**: The examiner assesses COWS-rating.
- **Time 167 hrs 35''** to **167 hrs 45''**: Identical assessments as made at baseline are repeated. A **red** marker is used to demarcate the secondary hyperalgesia area.
- **Time 167 hrs 45'' to 168 hrs 10''**: **Second** step TCI (including a bolus dose) of naloxone/placebo infusion (Fig. 4).
- **Time 167 hrs 57''** to **59''**: The examiner assesses COWS-rating.
- **Time 167 hrs 59''** to **168 hrs 09''**: Identical assessments as made at baseline are repeated. A **green** marker is used to demarcate the secondary hyperalgesia area.
- **Time 168 hrs 10''** to **168 hrs 35''**: **Third** step TCI (including a bolus dose) of naloxone/placebo infusion (Fig. 4).
- **Time 168 hrs 23''** to **168 hrs 25''**: The examiner assesses COWS-rating.
- **Time 168 hrs 25''** to **168 hrs 35''**: Identical assessments as made at baseline are repeated. A **black** marker is used to demarcate the secondary hyperalgesia area.
- **Time 168 hrs 35''**: End of naloxone/placebo infusion.
- Monitoring data are continuously registered and documented.
- Potential side-effects are continuously registered and documented
- **Time 168 hrs 35''** to **170 hrs 20''**: observation period where the participant is allowed to drink, eat and begin to ambulate. Monitoring is continued until **169 hrs 00''**. The peripheral i.v. cannula is removed at **169 hrs 00''**.
- **Time 170 hrs 20''**: the participant is discharged

The participants will be contacted by phone (AW) the day after each examination day to check up on potential side effects. The examiner (MUW) will be on call on mobile at the request of the participant for 24 hrs after each examination day.

9.2 Clinical assessments (TME-participants and BI-participants)

9.2.1 Psychometric evaluation

On Day 1 for TME-participants and Day 0 for BI-participants, a psychometric evaluation with HADS and PCS will be performed. These evaluations are made since anxiety, depressive thoughts and catastrophizing behavior may predispose to increased or even exaggerated pain sensitivity.

The Hospital Anxiety and Depression Scale (HADS) is used to assess anxiety and signs of depression. Based on 14 questions about the subject's status in the previous week, HADS measures agitation/anxiety and depression via two subscales (each containing seven questions). Participants have to answer each question on a scale of 0 to 3. The two subscales are summed separately. The maximum score of each subscale is 21 points and a score of 11 or more points suggests that the participant might be suffering from anxiety or depression. In case of score ≥ 11 points in the depression subscale of the HADS, a physician will decide if there are clinical signs of depression. If there are signs of depression, this diagnosis will be told to the participant. The participant will be informed that the diagnosis of depression is based on clinical assessments – the HADS scale can be included in the diagnostic procedure. If it is the participants wish, he should visit his general practitioner for diagnosis and eventual treatment. Participants with significant signs of depression will be excluded from the study.

The Pain Catastrophizing Scale (PCS) consists of 13 questions divided into three sections: rumination, exaggeration and helplessness. The questions are answered in accordance to a scale of 0 to 4. There is evidence of catastrophizing thoughts at a total score > 30 points.

9.2.2 Evaluation of opioid withdrawal (BI-participants Day 0 not included)

The Clinical Opiate Withdrawal Scale (COWS) is an examiner-based scale evaluating signs of opioid-withdrawal. Grading of symptoms, i.e. heart rate changes, sweating, restlessness, pupil size, bone or joint aches, running nose or tearing, nausea, vomiting, diarrhea, tremor, yawning, anxiety or irritability and “goose-flesh", are made in 11 categories. COWS-scores are divided into: 5-12 = mild; 13-24 = moderate; 25-36 = moderately severe ;> 36 = severe withdrawal reactions.

9.2.3 Reaction time

Online Reaction Time is measured using http://getyourwebsiteherecom/jswb/rttest01.htm. This computer-application shows a red-green traffic light. Participants are instructed to press the button when the light changes from red to green. Three measurements are used and the median value is used as a representative estimate of reaction time.

9.2.4 Pain assessments

9.2.4.1 TME-participants

Pain assessments are performed in three different situations:

* Pain rating (0-10, NRS) at rest in the supine position

* Pain rating (0-10, NRS) during a standardized mastication procedure. Pain is evaluated by asking the participant to chew a prepared[D] chewing gum (between the upper and lower, first molar of the surgical side during 30 seconds at a cycles of 40 Hz controlled by a iPhone-driven metronome (app. Steinway

D

Musical Instruments 1.1.4). Volunteers will be instructed that each chewing cycle ends when the upper and lower teeth clench and this time-point should be synchronized with the metronome. Volunteers will then rate the maximum pain intensity evoked by the task.

The sugar-free chewing gum (V6; Mondelez, Denmark) is prepared by the participant by chewing on the gum in 2" immediately at the start of the examination day. The gum is thereafter kept in a plastic container and re-used by the participant during the mastication procedure.

***** Pain rating (0-10, NRS) during a pressure stimulus (100 kPa) applied perpendicularly at the mandibular skin sites overlying the surgical area and the contralateral mirror-site, using a pressure algometer (Somedic AB, Hörby, Sweden) with a 1-cm^2 felt-tipped probe, directed at the point of maximum pain. During the measurement, the investigator will stabilize the lower jaw by sliding the gloved, index finger along the internal side of the body of the mandible, while applying lateral indirect pressure (100 kPa), externally to the relaxed masseter muscle. The participants rate the maximal, experienced pain intensity (0-10 NRS).

9.2.4.2 BI-participants

Pain ratings is assessed by NRS (0-10) at 0',30',60',120',180',240',300' and 360' after start of BI-induction.

9.2.5 Secondary hyperalgesia/allodynia

9.2.5.1 TME-participants

Secondary hyperalgesia/allodynia at surgical site and contralateral mirror-site at the mandibular skin sites overlying the surgical area and the contralateral mirror-site using a nylon monofilament (nominal value 4.93 [bending force: mean + SD = 69 + 14 mN]).

9.2.5.2 BI-participants

The secondary hyperalgesia area is determined using a “weighted-pin” stimulator (PinPrick stimulators, MRC Systems GmbH, Heidelberg, Germany; 128 mN [2,606 kPa]). The borders of the areas are assessed by stimulating along eight symmetrical lines, converging towards the center of the BI. The stimulation path starts at least 12 cm outside the borders of the BI-area. The participants are instructed to report, with their eyes closed, when the perception of the stimulus changes from an innocuous pin-prick to a stinging, smarting and unpleasant sensation, and the position is indicated by a marker. Normally, a secondary hyperalgesia area is **not** present in uninjured skin. If the participant anyhow experiences a transition zone, the participant is told to memorize the perceptual quality of the transition zone, and compare this with the perceptual quality during demarcation of secondary hyperalgesia following the burn injury (as a general rule there are clear perceptual qualitative and quantitative differences between hyperalgesia in an un-injured vis-á-vis an injured area. If the participant is unable to tell the difference, the participant is excluded from the study.

9.2.6 Pressure pain threshold (TME-participants)

Pressure pain threshold (PPT) will be assessed using an electronic pressure algometer (Algometer, Somedic AB, Sweden). The pressure algometer employs a 1-cm^2 circular probe, which is applied manually, perpendicularly to the skin with increasing pressure at a rate of 1-2 kPa/s. The assessment area will include the point of maximum pain. PPT assessments will be performed at baseline (-20 min to -8 min; Fig. 2) and during infusions (15 min to 25 min; 39 min to 49 min; 65 min to 75 min) at the surgical site and at the mirror-site in the contralateral groin. The participants interrupt the stimulation by pushing a button when the pressure becomes painful, and the investigator then stops the stimulation. The cut-off value is 350 kPa. Values exceeding the cut-off are designated 351 kPa.

9.2.7 Pin-prick pain threshold (BI-participants)

Pin-prick pain thresholds (PPTs) in the primary BI-area and in the secondary hyperalgesia area are assessed by "weighted-pin" stimulators (8 to 512 mN [81 to 10,424 kPa]), starting with the lightest pin-prick stimulator (8 mN), and applying a modified Dixon's up-and-down technique. Participants are instructed to report the number of stimuli perceived as unpleasant after a total of five pin-prick stimuli. The lowest pinprick force associated with ≥ 3 unpleasant stimuli is defined as the PPT. The median value of the five PPTs is used in further data analysis. The PPT-data (8, 16, 32, 64, 128, 256, 512 mN) are converted to corresponding ordinal values 1 to 8, where the "cut-off" value of 8 is registered if no pain is elicited during assessment with the heaviest pin-prick stimulator (512 mN).

10. STUDY DRUG AND PHARMACY PROCEDURE

10.1 Study drug (all participants [except for 10.1.2 only for TME-participants])

10.1.1 Naloxone

Naloxone 4 mg/ml is delivered in glass ampoules of 100 ml (total content 400 mg). Naloxone is dissolved in a 0.9% NaCl solution: 1 liter of solution contains 9 grams of sodium chloride in sterile water (sodium-chloride 154 mmol/l; osmolarity 308 mmol/l). The solutions are identical in regard to color, density, transparency and odor.

10.1.2 Placebo

Placebo is normal saline delivered in glass ampoules of 100 ml. One liter of placebo solution contains 9 g of sodium chloride in sterile water (sodium-chloride 154 mmol/l; osmolarity 308 mmol/l).

10.1.3 Rescue drugs

As rescue-analgesic the rapid-onset, opioid alfentanil, will be administered, which in less than one min completely will antagonize the effects of naloxone. Alfentanil is a well-known drug used in anesthesia for immediate relief of acute pain. Alfentanil is initially given 7-15 mikrog/kg (1-2 ml/70 kg) and is administered in a titrated fashion until the desired analgesic effect is achieved.

Other drugs that the investigator considers necessary for the management of adverse effects (adrenaline, atropine, midazolam, ondansetron) will be administered. The administration of rescue drugs will be recorded in the participant's Case Report Form.

10.2 Blinding procedure, packing and labeling

10.2.1 TME-participants and BI-participants

This is a cross-over, block-randomized, double-blind study. Randomization (computer generated using Randomization.com; sequence-randomization in blocks of four subjects), and, manufacturing, packaging and labelling of drugs will be performed by Skanderborg Hospital Pharmacy (att. Pharmacist Anna-Maria Simonsen). Two sets of non-transparent sealed envelopes with the complete randomization list will be prepared. These contain information on which treatment each participant participating in the study will receive. The sponsor stores one of the envelopes, while the other one is kept by the investigator at the Department of Anesthesiology HOC, 4231 Rigshospitalet in a secure locked place. The Skanderborg Hospital Pharmacy keeps the un-blinded randomization list, which will be sent to the investigator for data analysis after the study is finished. Individual drug-packages, for each participant, containing two 100 ml ampoules of normal saline and naloxone 4 mg/ml, respectively, include a sealed, non-transparent envelope containing the randomization sequence code, to be broken in case of a medical emergency (paragraph 12.4, page 29).10.2.2 Manufacturing, packaging and labelling

Manufacturing, packaging and labelling of drugs will be performed by Skanderborg Hospital Pharmacy (att. Pharmacist Anna Marie Simonsen).

10.3 Drug storage and accounting (all participants)

The investigators are responsible for safe storage of the study drug and are accountable that the drug is solely provided to participants of this study. The investigators will supervise the balancing and accounting of the study drugs. The investigator is responsible for explaining and document any loss of study drug, and, in particular if there is any discrepancy between the received quantity and returned quantity of drugs.

11. DRUG ADMINISTRATION

11.1 Target-controlled-infusion (all participants)

The target-controlled-infusion (TCI) algorithm (Fig. 2) was calculated by the software NONMEM (7.3 ICON Development Solutions, Manchester, U.K. [property of UCSF, U.S.]), using computer simulations based on a population-kinetic model with 2,000 simulated administrations distributed on 10 subjects (Table 1). Thus a total dose of 3.25 mg/kg of naloxone will be administered in a step-wise approach for 75 minutes as follows (Table 1): In the first minute (0-1 min) a bolus of 0.02 mg/kg will be given followed by an infusion of 0.23 mg/kg during 24 minutes (1-25 min). Afterwards (25-26 min) a bolus of 0.06 mg/kg will be administered followed by a 24-minute infusion of 0.69 mg/kg (min 26-50). Finally a bolus of 0.18 mg/kg (50-51 min) will be given followed by a 24-minute infusion of 2.07 mg/kg (51-75 min). With this administration algorithm estimated naloxone plasma concentrations (median [95% limits]) of 344 ng/ml [130;567], 1,059 ng/ml [400;1,752] and 3,196 ng/ml [1,205;5,276] at time intervals 15-25 min, 40-50 min and 65-75 min, respectively, are achieved (Fig. 2). For TME-participants an identical infusion-algorithm will be used for the placebo infusion. All outcomes for TME-participants will be assessed before infusion and 3 times during infusion at the above mentioned

time intervals. In BI-participants blood-samples for pharmacokinetic outcomes are obtained at specified time-points during and after the TCI (cf. 9.1.2).

	time (min)	dose (mg/kg)	dose/70 kg (mg)	volume/70 kg (ml)	ml/min/70 kg
**Bolus 1**	**0-1**	0.02	1.40	0.35	0.35
**Infusion 1**	**1-25**	0.23	16.10	4.03	0.17
**Bolus 2**	**25-26**	0.06	4.20	1.05	1.05
**Infusion 2**	**26-50**	0.69	48.30	12.08	0.50
**Bolus 3**	**50-51**	0.18	12.60	3.15	3.15
**Infusion 3**	**51-75**	2.07	144.90	36.23	1.51
**TOTAL**		3.25	227.50	56.88	

Table 1. Naloxone (4 mg/ml) i.v.-administration: timeline, dose/kg, dose/70 kg, volume/70 kg and infusion-rates/min/70 kg for the three step target-controlled-infusion. The total naloxone-dose administered is 227.5 mg per 70 kg BW. The total naloxone volume is 56.9 ml per 70 kg BW.

11.2 Infusion pump

11.2.1 TME-participants

Naloxone (3.25 mg/kg) or placebo will be administered i.v. on Day 1 and Day 2, according to the randomization procedure. A syringe (IVAC©) will be filled with a 100 ml solution of either naloxone 4 mg/ml or placebo and inserted into the pump (IVAC® P7000 syringe based pump).

11.2.2 BI-participants

Naloxone (3.25 mg/kg) will be administered i.v. on the study day. A syringe (IVAC©) will be filled with a 100 ml solution of naloxone 4 mg/ml and inserted into the pump (IVAC® P7000 syringe based pump).

12. SIDE EFFECTS

12.1 Side effects/Adverse events (AEs)

Side effects are defined as any harmful and unwanted event, sign or symptom, which occurs during the participation in the study and which is time related with the administration of the study drug, regardless of being considered to be caused by the study drug or not. All adverse events will be recorded in the participants' Case Report Form. If an adverse event occurs more than 6 hours after the last drug administration and there is no apparent causal connection or association with the study drug, it will not be considered an adverse event.

Start and end date & time, severity and impact of all the adverse events must be registered. The severity of the adverse event and its relationship to the study drug must be evaluated in accordance with the guidelines below.

Guidelines for classification of Adverse Events possibly related to the study

1. **Not related** – there is no temporal relationship; other etiologies are very likely.
2. **Possibly related** – correlation between study drug administration and adverse events is less clear; other etiologies are also possible.

3. **Probably related** – there is a clear temporal relationship between study drug administration and adverse events with improvement of the adverse event after discontinuing the study drug; participant's clinical condition does not explain the adverse event reasonably.
4. **Related** – there is a clear temporal relationship between study drug administration and adverse events.

Participants, experiencing adverse events, will be monitored with appropriate clinical evaluation and laboratory tests as indicated by the responsible physician. All participants with adverse events will be followed until satisfactory recovery or stabilization are attained.

A Serious Adverse Event (SAE) is not dose-dependent and results in a significant risk of death or disability for the participant, including, but not limiting itself, to an event that:

1. causes death.
2. is life-threatening - the participant was at imminent risk of dying from the adverse event, according to the investigator’s assessment.
3. requires hospitalization or prolongs already occurring hospitalization.
4. causes permanent disability.

The Summary of Product Characteristics of Naloxone published at the homepage of Danish Health and Medicines Authority will be used as a reference document, in case a serious adverse event has to be evaluated as expected or non-expected.

12.2 Grading of adverse events

The medical investigator will attempt to identify all clinical and objective adverse events and establish their relationship to the study drug. Adverse events, if any, should be graded according to the following scale:

1 = slight

2 = moderate

3 = severe

4 = life-threatening

12.3 Reporting of AEs and SAEs

The investigator has the responsibility to ascertain that all adverse events will be recorded and appropriately documented in the participant's Case Report Form. The Sponsor is responsible for the continuous evaluation of the study risk/benefit ratio. If a situation arises, that may affect the participants’ safety or that affects the ethical integrity of the study, it must be immediately reported to the Danish Health and Medicines Authority. A similar report must be sent to all the involved investigators and to the regional Research Ethical Committee.

Furthermore, the following rules for reporting to authorities should be taken into account. Any significant side-effect caused by drugs given in the study – the experimental treatment, comparison

drugs or placebo – must be reported. The sponsor has to be kept informed by the investigator of adverse events. The final report of the study should contain a description of all observed side effects.

SAEs must be reported immediately by the investigator to the sponsor and in addition a report on participants' safety should be sent once per year to the Research Ethical Committee.

Serious adverse reactions (SARs) (Serious, suspected adverse reactions), and in addition a report on participants safety, should be reported by the sponsor once per year to the Danish Health and Medicines Authority.

The sponsor must immediately report unexpected and **serious suspected adverse events** (SUSAR) to the Danish Health and Medicines Authority. Summary of Product Characteristics of naloxone will be used as a reference document for evaluating whether a serious related adverse reaction is unexpected or expected. **Fatal or life-threatening SUSAR's** must be reported to the Danish Health and Medicines Authority with all relevant information including follow-up within 7 days after the sponsor became aware of them, and no later than 8 days after notification. All other unexpected and serious suspected adverse events should be reported to the same authorities within 15 days after the sponsor has become aware of them. Reports should be accompanied by comments on the consequences for the study. It is also recommended that the sponsor informs the drug manufacturer.

12.4 Procedures in case of emergency

The investigator will ensure that there are planed procedures and the necessary expertise to deal with an emergency situation that may arise during the study. The randomization code (and thus the blinding procedure) should only be broken, if it is necessary to know which drug has been administered to the participant. Individual drug-packages include a sealed, non-transparent envelope containing the randomization sequence code for each participant (paragraph 10.2.1, page 26). This envelope is immediately broken by the investigator should an emergency arise that require un-blinding of the drug. If the code envelope is opened, the date and justification must be registered. The envelope must then be signed by the investigator and the participant will be excluded from the study.

13. STATISTICAL ANALYSES

13.1 Sample size calculations

13.1.1 TME-participants

Sample size calculations are based on following assumptions and estimations (abbreviations: BL = baseline; NX = naloxone; PL = placebo; TCI = during target-controlled-infusion):

1. a primary outcome based on a summed measure (SM) of resting pain (RP), masticatory pain (MP; (movement-related) and pressure-evoked pain (PM; pressure algometry [100 kPa]). $NRS_{SM} = NRS_{RP} + NRS_{MP} + NRS_{PM}$.

2. the summed pain intensities (SPIs) are calculated as: ($BL\text{-}SPI_{NX}$) and ($TCI\text{-}SPI_{NX}$), and, ($BL\text{-}SPI_{PL}$) and ($TCI\text{-}SPI_{PL}$), where (TCI-SPI) indicates SM-value at highest obtainable TCI-step (Fig. 2).

3. the summed pain intensity differences (SPIDs) are the differences: $\mathbf{SPID_{NX}}$ = ($TCI\text{-}SPI_{NX}$) – ($BL\text{-}SPI_{NX}$) and $\mathbf{SPID_{PL}}$ = ($TCI\text{-}SPI_{PL}$) - ($BL\text{-}SPI_{PL}$)

4. the mean [SD] BL-pain intensities are estimated to be: resting pain 1 [1] NRS-units; movement related pain 2 [2] NRS-units; pressure evoked pain 4 [2] NRS-units.

5. the mean [SD] summed BL-pain intensity (BL-SPI) is estimated to be 7 [4.1] NRS-units (Table 2).

6. the minimal relevant SPID-difference, calculated as **ΔSPID = $\mathbf{SPID_{NX}}$ - $\mathbf{SPID_{PL}}$** is estimated to be 5 (NX-group: RP = 4; MP = 5; PM = 7 [total 16]/PL-group: RP = 2; MP = 4; PM = 5 [total 11]) corresponding to a ΔSPID% = 45%.

7. the design is a placebo-controlled, randomized, double-blind, crossover study

8. the significance level α is set to 0.01 and the power is set to = 0.90 (β = 0.10)

The total number of individuals estimated to be included in the study is calculated[E] to be 14 (effect size 1.2). Using the range of variances in Table 2, it can be calculated that the number of individuals ranges from 9 to 19, with effect sizes of 1.7 to 1.0, respectively.

In order to allow for drop-outs the total number of individuals is set to 18. Due to the frail nature of the sample size estimates, an interim-analysis of the primary outcome, performed by an independent statistician, is made after per-protocol inclusion of 14 individuals. If the null hypothesis (no statistical difference between the placebo and the naloxone groups) is rejected, the study is deemed completed. If the new sample size estimates indicate that more than 16 additional individuals (total number of individuals > 30) are required, the study is prematurely discontinued. If less than 16 additional individuals (total number of individuals $\leq$ 30) the study continues and is discontinued after the stipulated number has been reached.

V(X1)	V(X2)	V(X3)	COV(X1,X2)	COV(X1,X3)	COV(X2,X3)	V(SUM)	SD(SUM)		CORR(X1,X2)	CORR(X1,X3)	CORR(X2,X3)
1	4	4	0	0	0	**9**	**3.00**		0	0	0
1	4	4	0.1	0.1	0.2	**9.8**	**3.13**		0.05	0.05	0.05
1	4	4	0.2	0.2	0.4	**10.6**	**3.26**		0.1	0.1	0.1
1	4	4	0.3	0.3	0.6	**11.4**	**3.38**		0.15	0.15	0.15
1	4	4	0.4	0.4	0.8	**12.2**	**3.49**		0.2	0.2	0.2
1	4	4	0.5	0.5	1	**13**	**3.61**		0.25	0.25	0.25
1	4	4	0.6	0.6	1.2	**13.8**	**3.71**		0.3	0.3	0.3
1	4	4	0.7	0.7	1.4	**14.6**	**3.82**		0.35	0.35	0.35
1	4	4	0.8	0.8	1.6	**15.4**	**3.92**		0.4	0.4	0.4
1	4	4	0.9	0.9	1.8	**16.2**	**4.02**		0.45	0.45	0.45
1	4	4	1	1	2	**17**	**4.12**		0.5	0.5	0.5
1	4	4	1.1	1.1	2.2	**17.8**	**4.22**		0.55	0.55	0.55
1	4	4	1.2	1.2	2.4	**18.6**	**4.31**		0.6	0.6	0.6
1	4	4	1.3	1.3	2.6	**19.4**	**4.40**		0.65	0.65	0.65
1	4	4	1.4	1.4	2.8	**20.2**	**4.49**		0.7	0.7	0.7
1	4	4	1.5	1.5	3	**21**	**4.58**		0.75	0.75	0.75
1	4	4	1.6	1.6	3.2	**21.8**	**4.67**		0.8	0.8	0.8
1	4	4	1.7	1.7	3.4	**22.6**	**4.75**		0.85	0.85	0.85
1	4	4	1.8	1.8	3.6	**23.4**	**4.84**		0.9	0.9	0.9
1	4	4	1.9	1.9	3.8	**24.2**	**4.92**		0.95	0.95	0.95
1	4	4	2	2	4	**25**	**5.00**		1	1	1

Table 2. The variance (V) of the summed pain intensity (SPI) is:
$V_{SPI} = V_{RP} + V_{MP} + V_{PM} + 2*(COV_{(RP,MP)} + COV_{(RP,PM)} + COV_{(PM,MP)})$
However, the covariances are unknown, but the table illustrates the relationship between V_{SPI} (blue column; SD_{SPI} green column) vis-á-vis different correlations between the variables (RP, MP, PM). A correlation estimate between the variables of 0.5 was used.

[E] G*Power version 3.9.1.2, University of Kiel, Germany

13.1.2 BI-participants

Data from our previous high-dose naloxone study (Pereira MP et al [2015] PLOS One 10: e0134441. 10.1371/journal.pone.0134441 [doi]) indicate:

1. for the high-sensitizers (n = 3): secondary hyperalgesia areas 168 hrs after the BI during placebo infusion, mean (SD), 2.1 cm^2 (2.5 cm^2) and during naloxone infusion 111.0 cm^2 (26.3 cm^2).

2. for the low-sensitizers (n = 3): secondary hyperalgesia areas 168 hrs after the BI during placebo infusion, 0.3 cm^2 (0.1 cm^2) and during naloxone infusion 0.9 cm^2 (0.6 cm^2).

Using the significance level α set to 0.01 and the power set to = 0.90 (β = 0.10), the estimated number of individuals needed to reject the null hypothesis in the high-sensitizers are 5 (effect size 4.1; sic!) and in the low-sensitizers are 18 (effect size 1.4). Since the data used for sample size estimates are made on an extremely limited number of individuals, and, since the study of nature is a proof-of-concept, it was decided to include 20 high-sensitizers and 20 low-sensitizers. Boot-strapping techniques could have been used, but we considered the number individuals used in the sample estimate too extreme. On the other hand, if a larger number of subjects are needed, the clinical significance is deemed of dubious value.

13.2 Data processing

13.2.1 TME-participants

To test if data is normally distributed, residual plots and the Kolmogorov-Smirnov test will be used (SPSS 20.0, Chicago, IL, USA; MedCalc Software: version 12.07.0.0; Mariakerke, Belgium). In case of non-normal data distribution, primarily a logarithmic transformation is tried or secondarily a Box-Cox transformation will be attempted for normalization of data.

The primary outcome ΔSPID ($SPID_{NX}$ - $SPID_{PL}$) is analyzed by one-way repeated measures ANOVA (ΔSPID: dependent variable; TCI-steps: independent variable) or a corresponding non-parametric analogue (Friedman test), as appropriate. Paired *t*–test or Wilcoxon rank sum test will be used, as appropriate for inter-group comparisons.

A mixed model with random effect for subject and fixed-effects for the factors; *target-controlled-infusion* (step 1/step 2/step 3), *drug* (naloxone/placebo); *secondary hyperalgesia area* (SHA); *pressure pain threshold* (PPT); *HADS-score*; and *PCS-scores*, are used for the primary outcome ΔSPID ($SPID_{NX}$ - $SPID_{PL}$). Non-significant ($P > 0.05$) factors, beginning with interactions, are excluded until all included factors attain significance. Main-effects and interaction-effects are examined.

The risk of type I error is reduced by setting a significance level of 0.01 (α). A power of 0.90 (β = 0.10) is set in order to reduce the risk of type II errors. For all statistical calculations, in which multiple comparisons are performed, Duncan's new multiple range test or Scheffé's method, as appropriate, will be used. Statistical calculations will be performed with partially un-blinded data, i.e., group A and B. When statistics have been completed, data are unblinded, into naloxone and placebo groups.

13.2.1 BI-participants

To test if data is normally distributed, residual plots and the Kolmogorov-Smirnov test will be used (SPSS 20.0, Chicago, IL, USA; MedCalc Software: version 12.07.0.0; Mariakerke, Belgium). In case of non-normal data distribution, primarily a logarithmic transformation is tried or secondarily a Box-Cox transformation will be attempted for normalization of data.

The differences calculated are:

$\Delta SHA_{high\text{-}sensitizers} = SHA_{NX\ high\text{-}sensitizers} - SHA_{PL\ high\text{-}sensitizers}$ (max. values during one of the 3 TCI-steps)

$\Delta SHA_{low\text{-}sensitizers} = SHA_{NX\ low\text{-}sensitizers} - SHA_{PL\ low\text{-}sensitizers}$ (max. values during one of the 3 TCI-steps)

$\Delta SHA_{sensitizers} = \Delta SHA_{high\text{-}sensitizers} - \Delta SHA_{low\text{-}sensitizers}$

A mixed model with random effect for subject and fixed-effects for the factors: *secondary hyperalgesia areas* (SHA); *sensitizers* (high-sensitizers/low-sensitizers); *target-controlled-infusion* (step 1/step 2/step 3); *HADS-scores*; and *PCS-scores*, are used for the primary outcome $\Delta SHA_{sensitizers}$. Non-significant ($P > 0.05$) factors, beginning with interactions, are excluded until all included factors attain significance. Main-effects and interaction-effects are examined.

The risk of type I error is reduced by setting a significance level of 0.01 (α). A power of 0.90 ($\beta = 0.10$) is set in order to reduce the risk of type II errors. For all statistical calculations, in which multiple comparisons are performed, Duncan's new multiple range test or Scheffé's method, as appropriate, will be used. Statistical calculations will be performed with partially un-blinded data, i.e., group A and B. When statistics have been completed data are unblinded into naloxone and placebo groups.

14. DATA REGISTRATION

14.1 Data registration and control of investigation procedures

The study will be conducted in accordance with the guidelines and rules concerning quality control and quality management on clinical trials involving humans, and will follow the GCP and GMP guidelines. The investigator, affiliated to Rigshospitalet, is responsible for managing and archiving the study data according to the current regulations including regulations on personal data processing and regulations on health care. Data belongs to the sponsor/principal investigator Mads U. Werner and assistant investigator Anne Willum. The study is reported to the Data Protection Agency.

14.2 Case Report Form

For each participant included in the study, a Case Report Form (CRF) will be filled out. The CRFs will be signed by the investigator to confirm the accuracy of the data. Corrections of data will be performed by cross-lining the incorrect data (the incorrect data will remain visible and legible) and the correct data will be written next to the cross-lined data. Correction fluid will not be applied. The investigator or other staff member involved in the study will date and sign the corrections. Source data is the CRF.

14.3 GCP-Monitoring

GCP unit – Copenhagen University Hospitals

Bispebjerg Hospital, Building 51, 3.

Bispebjerg Bakke 23

2400 København NV

Phone: +45 3531 3890

15. Miscellaneous

15.1 Insurance

For participants in this study it is, with respect to any damage caused directly or indirectly by the study medication in this clinical study at the Department of Anaesthesiology 4231, HOC Rigshospitalet, is on the responsibility of the investigator and his staff's behalf, provided the investigator and his staff have followed the instructions given in this protocol and any amendments. Furthermore that, the investigator and his staff have conducted the study scientifically and in accordance with applicable rules and accepted techniques. Participants in the event of injury or death unrelated to the conduct of the study insured by hospital insurance.

15.2 Financial issues

The study is partially funded by National Institute of Health (NIH-NIDA) NIH R01DA037621. Additional grants will be sought from private funds. A preliminary budget is indicated below:

	USD	DKR
Year	1	1
Research Associate Months	5	
Research Associate's Salary	$ 28,762	198,168
Research Assistant Months	5	
Research Assistant's Salary	$ 7,553	52,037
Volunteers' salaries	$ 16,546	114,000
Patients' Reimbursement TME	$ 2,351	16,200
Maintenance QST Equipment	$ 2,599	17,907
Drug costs	$ 9,289	64,000
Miscellaneous Lab. Costs	$ 1,109	7,638
Compliance cost to Rigshospitalet Copenhagen (8%)	$ 4,948	34,092
**Total**	**$ 73,156**	**504,041**

15.3 Publication of Results

Positive, negative or inconclusive results will be published. A report based on the data of the study will be prepared by the investigators. The report will be forwarded to the relevant authorities and will be the basis of two manuscripts submitted for publication in an international scientific journal with the following order of authors:

1. *TME-study:* Werner MU, Willum A, Springborg A, surgeon, Kreilgaard M, Taylor BK.
2. *BI-study*: Springborg A, Taylor BK, Willum A, Werner MU.

Clinical Opiate Withdrawal Scale (COWS)

Flow-sheet for measuring symptoms over a period of time during buprenorphine induction.

For each item, write in the number that best describes the patient's signs or symptom. Rate on just the apparent relationship to opiate withdrawal. For example, if heart rate is increased because the patient was jogging just prior to assessment, the increase pulse rate would not add to the score.

Patient's Name:__________________________ Date: _____________
Buprenorphine induction:
Enter scores at time zero, 30min after first dose, 2 h after first dose, etc.
Times: _____ _____ _____ _____

**Resting Pulse Rate**: (record beats per minute)<br>*Measured after patient is sitting or lying for one minute*<br>0 pulse rate 80 or below<br>1 pulse rate 81-100<br>2 pulse rate 101-120<br>4 pulse rate greater than 120				
**Sweating:** *over past ½ hour not accounted for by room temperature or patient activity.*<br>0 no report of chills or flushing<br>1 subjective report of chills or flushing<br>2 flushed or observable moistness on face<br>3 beads of sweat on brow or face<br>4 sweat streaming off face				
**Restlessness** *Observation during assessment*<br>0 able to sit still<br>1 reports difficulty sitting still, but is able to do so<br>3 frequent shifting or extraneous movements of legs/arms<br>5 Unable to sit still for more than a few seconds				
**Pupil size**<br>0 pupils pinned or normal size for room light<br>1 pupils possibly larger than normal for room light<br>2 pupils moderately dilated<br>5 pupils so dilated that only the rim of the iris is visible				
**Bone or Joint aches** *If patient was having pain previously, only the additional component attributed to opiates withdrawal is scored*<br>0 not present<br>1 mild diffuse discomfort<br>2 patient reports severe diffuse aching of joints/ muscles<br>4 patient is rubbing joints or muscles and is unable to sit still because of discomfort				
**Runny nose or tearing** *Not accounted for by cold symptoms or allergies*<br>0 not present<br>1 nasal stuffiness or unusually moist eyes<br>2 nose running or tearing<br>4 nose constantly running or tears streaming down cheeks				

**GI Upset**: *over last ½ hour*<br>0 no GI symptoms<br>1 stomach cramps<br>2 nausea or loose stool<br>3 vomiting or diarrhea<br>5 Multiple episodes of diarrhea or vomiting				
**Tremor** *observation of outstretched hands*<br>0 No tremor<br>1 tremor can be felt, but not observed<br>2 slight tremor observable<br>4 gross tremor or muscle twitching				
**Yawning** *Observation during assessment*<br>0 no yawning<br>1 yawning once or twice during assessment<br>2 yawning three or more times during assessment<br>4 yawning several times/minute				
**Anxiety or Irritability**<br>0 none<br>1 patient reports increasing irritability or anxiousness<br>2 patient obviously irritable anxious<br>4 patient so irritable or anxious that participation in the assessment is difficult				
**Gooseflesh skin**<br>0 skin is smooth<br>3 piloerrection of skin can be felt or hairs standing up on arms<br>5 prominent piloerrection				
**Total scores**<br><br>**with observer's initials**				

Score:
5-12 = mild;
13-24 = moderate;
25-36 = moderately severe;
more than 36 = severe withdrawal

Vi er interesserede i, hvilke tanker og følelser du har, når du har smerter.
Nedenfor er tretten udsagn, der beskriver forskellige tanker og følelser, der kan høre sammen med det at have smerter. Ved brug af nedenstående tabel bedes du markere, i hvilken grad du har disse tanker og følelser, når du oplever smerte.

Når jeg har smerter...

		Slet ikke	I mindre grad	i moderat grad	i høj grad	hele tiden
a.	Jeg bekymrer mig hele tiden om, hvornår smerten stopper	□	□	□	□	□
b.	Jeg føler, at jeg ikke kan mere	□	□	□	□	□
c.	Det er forfærdeligt og jeg tror, at det aldrig bliver bedre	□	□	□	□	□
d.	Det er frygteligt, og jeg føler, det overvælder mig	□	□	□	□	□
e.	Jeg føler, jeg ikke kan holde det ud mere	□	□	□	□	□
f.	Jeg bliver bange for, at smerten skal blive værre	□	□	□	□	□
g.	Jeg bliver ved med at tænke på andre smertefulde hændelser	□	□	□	□	□
h.	Jeg er ivrig efter at få smerten til at gå væk	□	□	□	□	□
i.	Jeg kan ikke slå det ud af hovedet	□	□	□	□	□
j.	Jeg bliver ved med at tænke på, hvor ondt det gør	□	□	□	□	□
k.	Jeg bliver ved med at tænke på, hvor meget jeg ønsker, at smerten skal stoppe	□	□	□	□	□
l.	Der er intet, jeg kan gøre for at mindske, hvor kraftig smerten er	□	□	□	□	□
m.	Jeg tænker på, om der måske sker noget alvorligt	□	□	□	□	□

a. Jeg er anspændt eller stresset.

☐ Det meste af tiden ☐ Meget af tiden

☐ Engang imellem ☐ Overhovedet ikke

b. Jeg glæder mig stadig over de ting, jeg plejer at glæde mig over.

☐ Helt bestemt ☐ Ikke helt så meget

☐ Kun lidt ☐ Næsten ikke

c. Jeg får en slags skræmmende fornemmelse, som om noget frygteligt skal til at ske.

☐ Helt bestemt og temmelig slemt ☐ Ja, men ikke alt for slemt

☐ En smule, men det bekymrer mig ikke ☐ Overhovedet ikke

d. Jeg kan le og se tingene fra den morsomme side.

☐ Lige så meget som jeg altid har kunnet ☐ Ikke helt så meget nu

☐ Bestemt ikke så meget nu ☐ Overhovedet ikke

e. Bekymrende tanker strejfer mig.

☐ En meget stor del af tiden ☐ Meget af tiden

☐ Engang imellem, men ikke så tit ☐ Kun engang imellem

f. Jeg er i godt humør.

☐ Overhovedet ikke ☐ Ikke ofte

☐ Nogle gange ☐ Det meste af tiden

g. Jeg kan sidde roligt og føle mig afslappet.

☐ Helt bestemt ☐ For det meste

☐ Ikke ofte ☐ Overhovedet ikke

h. Jeg føler det som om, jeg virker sløv.

☐ Næsten hele tiden ☐ Meget ofte

☐ Somme tider ☐ Overhovedet ikke

i. Jeg får en slags bange fornemmelse, som "sommerfugle" i maven.

☐ Overhovedet ikke ☐ Ikke ofte

☐ Ret ofte ☐ Meget ofte

j. Jeg har mistet interessen for mit udseende.

☐ Helt bestemt ☐ Jeg er ikke helt så omhyggelig som jeg plejer at være

☐ Måske interesserer det mig knap så meget som før ☐ Jeg er lige så omhyggelig som før

k. Jeg føler mig rastløs, som om jeg hele tiden skal være i gang.

☐ I udtalt grad ☐ En hel del

☐ Ikke så ofte ☐ Overhovedet ikke

l. Jeg ser med glæde frem til tingene.

☐ Lige så meget, som jeg altid har gjort ☐ En del mindre end jeg plejer

☐ Bestemt mindre, end jeg plejer ☐ Næsten ikke

m. Jeg får pludselige fornemmelser af panik.

☐ Absolut meget ofte ☐ Temmelig ofte

☐ Ikke ret tit ☐ Overhovedet ikke

n. Jeg kan nyde en god bog, et radioprogram eller TV-program.

☐ Ofte ☐ Nogle gange

☐ Ikke ofte ☐ Meget sjældent
