# Supplementary material for: High-dose naloxone: Effects by late administration on pain and hyperalgesia following a human heat injury model. A randomized, double-blind, placebo-controlled, crossover trial with an enriched enrollment design
Source: PLoS One. 2020 Nov 12;15(11):e0242169. doi: 10.1371/journal.pone.0242169 (PMC7660513; doi:10.1371/journal.pone.0242169)
Supplement: S1 Adverse events — (DOCX) [file pone.0242169.s004.docx]

**S1 File. Adverse Events.**

**Events Related to Trial Drug**

Adverse effects of mild intensity including tiredness, nausea, vomiting, dizziness, headache, itching, and restlessness were experienced by 22/38 subjects receiving naloxone. Following the placebo infusion, four out of 38 subjects (Chi^2^ test: *P* < 0.0001) experienced mild adverse effects, including tiredness, headache, nausea, and itching. Two subjects experienced unpleasantness due to the IV cannula insertion procedure.

One subject (#AH39) in Session **2**, experienced anxiety, nausea, and dizziness concomitant with the observation of dilated pupils during the infusion. The infusion therefore was discontinued at the end of step 2 (min 40). The secondary hyperalgesia area (SHA) assessments in step 2 and step 3 as well as pin-prick pain threshold (PPT) assessments were performed after the discontinuation of the infusion. The subject subsequently expressed a desire to continue in the following experimental sessions (Session **3** and **4**) and was allowed further participation in the trial. Later at the end of the trial, un-blinding showed that the allocated infusion was naloxone.

Another subject (#AX25) experienced paresthesia of the arm, leg, and circumorally in addition to experiencing fear and dizziness during the infusion. The infusion was discontinued at the end of step 3 (min 70). The principal investigator administered anxiolytic 1.5 mg midazolam IV with good effect. The last SHA and PPT assessments were performed a few minutes after the discontinuation of the infusion. Later at the end of the trial, un-blinding showed that the allocated infusion was naloxone.

There was no significant difference between the number of subjects estimating the correct day the active drug was given (n = 25) *vs.*the number who did not (n = 13; binomial test: *P* = 0.073)

**Events Unrelated to Trial Drug**

One subject (#92) experienced a vasovagal syncope immediately following the cutaneous heat injury (CHI) during Session **0**. The subject was briefly unconscious, and experienced involuntary fecal incontinence. Subsequently, the subject was closely monitored by a physician for 2 h, until he was well enough to be discharged. A follow-up phone call was made the following day, and no additional symptoms were reported. No further assessments were made on this subject, and the subject was excluded from further participation in the trial.

**Events Unrelated to Trial**

One subject (#30) was diagnosed with acute lymphocytic leukemia shortly after inclusion in the experimental sessions and before receiving any trial intervention. The subject was discontinued in the trial, and the event was reported as a ‘serious adverse event not related to the trial’ to the Danish Medicines Agency. Another subject (#AJ04) attended an additional Session **3** due to illness on the initial day of Session **4**. The subject then participated in Session **4** one week after the second Session **3** as per-protocol.
